# No Detection of SARS-CoV-2 RNA on Urethral Swab in Patients with Positive Nasopharyngeal Swab

**DOI:** 10.1155/2020/8826943

**Published:** 2020-12-09

**Authors:** Lorenzo Spirito, Biagio Pinchera, Angela Patrì, Mario Delfino, Ciro Imbimbo, Paola Salvatore, Ivan Gentile, Gabriella Fabbrocini

**Affiliations:** ^1^Department of Neurosciences, Reproductive and Odontostomatological Sciences, Section of Urology, University of Naples Federico II, Naples, Italy; ^2^Department of Clinical Medicine and Surgery, Section of Infectious Diseases, University of Naples Federico II, Naples, Italy; ^3^Department of Clinical Medicine and Surgery, Section of Dermatology, University of Naples Federico II, Naples, Italy; ^4^Department of Molecular Medicine and Medical Biotechnology, University of Naples Federico II, Naples, Italy

## Abstract

**Background:**

The SARS-CoV-2 infection has caused one of the worst pandemics that history has ever known. SARS-CoV-2 can lead to multiple organ failure, which is life-threatening. Viral RNA is found in the lung, intestine, testicle, kidney, etc., which suggests the virus can be transmitted also via routes besides respiratory droplets. The aim of our study was to evaluate the presence of SARS-CoV-2 in urethral swabs.

**Methods:**

We enrolled ten patients with SARS-CoV-2 infection who attended the Infectious Diseases Unit of the A.O.U. Federico II of Naples, from March 2020 to April 2020. One urethral swab and one rhino-oropharyngeal swab were collected from each patient during SARS-CoV-2 infection.

**Results:**

All ten patients had a negative urethral swab for SARS-CoV-2 RNA, whereas the rhino-oropharyngeal swab was positive for SARS-CoV-2 RNA. This finding demonstrates that, in our patients, the virus did not affect the urinary tract and therefore would not be found in the urine, and even more importantly, it would not be transmitted via urine. This result was independent of the stage of the disease.

**Conclusion:**

If confirmed in larger studies, this observation could be the key to understanding the role of SARS-CoV-2 in relation to the genitourinary system.

## 1. Introduction

The recent SARS-CoV-2 infection is the cause of one of the most important pandemics that history has ever experienced. SARS-CoV-2 is the causative agent of COVID-19 [1, 2]. The main manifestations of COVID-19 infection are fever, dry cough, and asthenia. Rapid progression to acute respiratory distress syndrome, septic shock, metabolic acidosis, coagulation deficiency, and lastly multiple organ failure may occur in critically ill patients [3]. Most patients have a good prognosis, while few develop a severe disease. The virus can be transmitted from person to person, directly or indirectly, and via the respiratory, orofecal, and probably sexual routes [4, 5]. The rapid development and serious consequences of the infection prompted authorities to contain the pandemic and researchers to study the transmission pathways [6]. The human receptor of SARS-CoV-2 is angiotensin-converting enzyme 2, which is expressed in the lung, intestine, testicle, kidney, etc. Viral RNA has also been identified in faeces, blood, semen [7], tears [8], and urine. However, the mechanisms via which the infection is transmitted are not fully understood.

In addition, given the planetary nature of the pandemic, it is necessary to address such real-life issues as sexual behaviour. For example, is it safe for a patient with SARS-CoV-2 infection to have sexual contacts after a negative nasopharyngeal swab? And should Sars-CoV-2 be considered a sexually transmitted disease? The aim of our study was to evaluate the presence of SARS-CoV-2 in urethral swabs collected in 10 consecutive patients with COVID-19.

## 2. Materials and Methods

We enrolled patients with SARS-CoV-2 infection who consecutively attended the Infectious Diseases Unit of the A.O.U. Federico II of Naples from March 2020 to April 2020. SARS-CoV-2 RNA infection was defined by positivity of the rhino-oropharyngeal swab for SARS-CoV-2 RNA by reverse polymerase chain reaction (rRT-PCR). COVID-19 was defined by a positive rhino-oropharyngeal swab for SARS-CoV-2 RNA by reverse polymerase chain reaction (rRT-PCR) and by clinical-laboratory-instrumental manifestations characteristic of this infection. The clinical manifestations were fever, dry cough, asthenia, nasal obstruction, rhinorrhea, pharyngodynia, myalgia, diarrhea, dyspnea, hypoxemia, acute respiratory distress syndrome, septic shock, acidosis metabolic, coagulation deficiency, and multiple organ failure (MOF). As for blood chemistry tests, lymphopenia, elevated transaminases, lactate dehydrogenase (LDH), creatine kinase (CPK), and myoglobin, troponin T elevation (TnT), increased D-dimer, and an increase in reactive protein C (PCR) and erythro-sedimentation rate (ESR) were reported. In the initial phase of the disease, a nodular and/or interstitial pattern occurs, especially in the periphery of the parenchyma. Subsequently, frosted glass patterns develop bilaterally, infiltrative patterns [9]. In turn, patients with SARS-CoV-2 infection were distinguished by the stage of the disease; in particular, they distinguished patients with an asymptomatic form, patients with a mild-moderate form (symptoms, no pneumonia), patients with nonsevere pneumonia (patients with pneumonia and no signs of severe pneumonia and no need for oxygen therapy), patients with severe pneumonia (fever or suspected respiratory infection and at least one of the following: respiratory rate >30 acts/min and severe breathing difficulty or SpO_2_ <93% in ambient air), and patients with acute respiratory distress syndrome (ARDS) [10]. A urethral swab and a rectal swab were collected from each patient during SARS-CoV-2 infection. SARS-CoV-2 was identified by rRT-PCR. For patients who presented with a urethral swab and/or a rectal swab positive at the time of diagnosis, the swab was performed again at the time of the patient's virological recovery. Patients were considered virologically cured, when they had two negative rhino-oropharyngeal swabs for SARS-CoV-2 RNA, performed at least 24 hours apart [11]. Subjects with the following inclusion and exclusion criteria were enrolled.

Inclusion criteria were patients with a positive rhino-oropharyngeal swab for SARS-CoV-2 RNA; age >18 years; intention to participate in the study; and who provided informed consent. Exclusion criteria were age <18 years; inability to understand or sign informed consent; and any other condition that in the opinion of the experienced person could make the patient unsuitable for enrollment or that could interfere with the patient's participation in the study and its completion.

Nonparametric tests (Wilcoxon–Mann–Whitney test) were used to evaluate. Multivariate statistical analysis (principal components analysis, hierarchical clustering, principal coordinates analysis) were used to identify the groupings of the samples and the definition of the variables that allowed these groupings. All significance values are adjusted to take into account the effect of multiple comparisons, using the Benjamini–Hochberg correction, and a false discovery rate value <0.05 was considered statistically significant. A *p* value <0.05 was considered statistically significant for all tests conducted in the study.

## 3. Results

Ten patients were enrolled (7 men and 3 women) (see Table 1); of these, six had pneumonia (one severe), and the others had mild to moderate symptoms. Only one appeared to be asymptomatic. None of the patients were hospitalized in an intensive care unit. Eight were treated with hydroxychloroquine (200 mg 1 tablet* × * 2/day), lopinavir/ritonavir (200/50 mg 2 tablets* × *2/day), and azithromycin (500 mg 1 tablet/day). Treatment was administered for 14 days. Two patients received tocilizumab (8 mg/kg/day every 12 hours twice). Two patients did not receive therapy. All enrolled patients received low-weight heparin. All ten patients achieved clinical and virological cure. Seven of the 10 patients enrolled had comorbidities: 3 were suffering from cardiovascular disease, 1 from anxiety-depressive syndrome, 1 from colon adenocarcinoma, 1 from cognitive decline, and 1 from chronic pancreatitis. None of the patients had a history of disorders and/or pathologies attributable to the urinary genitourinary system. The urethral swab was negative for SARS-CoV-2 RNA in all ten patients during the active phase of the disease, i.e., when the rhino-oropharyngeal swab was positive for SARS-CoV-2 RNA.

## 4. Discussion

The pathophysiology of COVID-19 is still largely obscure, and similarly, data on SARS-CO-V2 RNA in the urethra are lacking. We did not detect the virus at urethral level. The virus has been detected in the ocular secretions, faeces, and seminal fluid of patients with COVID-19. These findings suggested that the transmission modalities of the SARS-CoV-2 may go beyond the respiratory route. Herein, we found that the virus was not found in urethral swab, which indicates that transmission via urine is unlikely. Notably, this result was independent of disease stage. In fact, regardless of the severity of the clinical conditions, all patients had a negative urethral swab for SARS-CoV-2 RNA during the active phase of the disease, i.e., when they were positive for SARS-CoV-2 RNA on rhino-oropharyngeal swab.

## 5. Conclusion

We acknowledge that our study has several limitations, for example, the small sample size and the use of urethral swab as the only site of detection. However, this is the first observation, which needs to be further investigated in larger studies, of the lack of detection of SARS-CoV-2 in urethral swabs from patients with COVID-19, during an active virological and clinical phase of the disease.

## Figures and Tables

**Table 1:** Anagraphic clinical features of enrolled patients.

Age (median, IQR)	64.9 (37–84)

Gender	
Male	7 (70%)
Female	3 (30%)

Urethral swab	
Positive for SARS-CoV-2 RNA	0 (0%)
Negative for SARS-CoV-2 RNA	10 (100%)

D-dimer (mg/l, median, IQR)	2.198 (0.29–6.72)

Fibrinogen (mg/dl, median, IQR)	566.7 (327–828)

C-reactive protein (mg/l, median, IQR)	30.8 (7–119)

Albumin (g/dl; median, IQR)	3.9 (3–4.6)

Platelets (elements/µL; median, IQR)	218,000 (178,000–560,000)

INR (median, IQR)	0.9 (0.8–1.2)

ALT (IU/l, median, IQR)	23 (18–27)

AST (IU/l, median, IQR)	21 (15–29)

Tot. Bil. (mg/dl; median, IQR)	0.8 (0.6–1.3)

Staging COVID-19	
Asymptomatic	1 (10%)
Mild to moderate symptoms	3 (30%)
Nonsevere pneumonia	5 (50%)
Severe pneumonia	1 (10%)

Antiviral therapy	
Hydroxychloroquine + lopinavir/ritonavir + azithromycin	8 (80%)
Tocilizumab	2 (20%)

## Data Availability

The data used to support the findings of this study are available from the corresponding author upon request.

## Abbreviations

SARS-CoV-2: Severe acute respiratory syndrome coronavirus 2
COVID-19: Corona virus disease 19.

## References

[1] Patrì A., Gallo L., Annunziata M. C., Megna M., Fabbrocini G. (2020). Corona virus disease-2019. COVID-19 pandemic: University of Naples Federico II Dermatology’s model of dermatology reorganization. *International Journal of Dermatology*.

[2] Abenavoli L., Cinaglia P., Luzza F., Gentile I., Boccuto L. (2020). Epidemiology of coronavirus disease outbreak: the Italian trends. *Reviews on Recent Clinical Trials*.

[3] Anelli F., Leoni G., Monaco R. (2020). Italian doctors call for protecting healthcare workers and boosting community surveillance during covid-19 outbreak. *BMJ*.

[4] Viceconte G., Petrosillo N. (2020). COVID-19 R0: magic number or conundrum?. *Infectious Disease Reports*.

[5] Johns Hopkins University *Center for Systems Science and Engineering: Coronavirus COVID-19 Global Cases*.

[6] Corman V. M., Landt O., Kaiser M. (2020). Detection of 2019 novel coronavirus (2019-nCoV) by real-time RT-PCR. *Euro Surveill*.

[7] Paoli D., Pallotti F., Colangelo S. (2020). Study of SARS-CoV-2 in semen and urine samples of a volunteer with positive naso-pharyngeal swab. *Journal of Endocrinological Investigation*.

[8] Colavita F., Lapa D., Carletti F. (2020). SARS-CoV-2 isolation from ocular secretions of a patient with COVID-19 in Italy with prolonged viral RNA detection. *Annals of Internal Medicine*.

[9] Centre for Disease Control and Prevention (2020). Information for clinicians on therapeutic options for COVID-19 patients. March 21, 2020, Wu Z, McGoogan JM. Characteristics of and important lessons from the coronavirus disease 2019 (COVID-19) outbreak in China: summary of a report of 72 314 cases from the Chinese Center for Disease Control and Prevention. *JAMA*.

[10] World Health Organization (2020). Clinical Management of Severe Acute Respiratory Infection (SARI) When COVID-19 Disease Is Suspected.

[11] Lombardia S. I. M. I. T. (2020). Vademecum per la cura delle persone con malattia da COVI-19 Edizione 2.0, 13 marzo 2020. *Clinical Management of Severe Acute Respiratory Infection (SARI) When COVID-19 Disease is Suspected*.

